# Role of ERK signaling in bladder urothelium in response to cyclophosphamide injury

**DOI:** 10.14814/phy2.15378

**Published:** 2022-07-19

**Authors:** Sridhar Tatarao Narla, Joanne Lindsey Duara, Daniel Scott Bushnell, Mehdi Nouraie, Jacqueline Holden, Katherine Pfister, Peter C. Lucas, Sunder Sims‐Lucas, Carlton Matthew Bates

**Affiliations:** ^1^ Department of Pediatrics, Division of Nephrology University of Pittsburgh School of Medicine Pittsburgh Pennsylvania USA; ^2^ Department of Pediatrics, Division of Neonatology University of Pittsburgh School of Medicine Pittsburgh Pennsylvania USA; ^3^ Department of Medicine Division of Pulmonary, Allergy and Critical Care Medicine, University of Pittsburgh School of Medicine Pittsburgh Pennsylvania USA; ^4^ Department of Pathology University of Pittsburgh School of Medicine Pittsburgh Pennsylvania USA; ^5^ Division of Nephrology UPMC Children's Hospital of Pittsburgh Pittsburgh Pennsylvania USA

**Keywords:** bladder, cyclophosphamide, ERK, fibroblast growth factor receptor 2, urothelium

## Abstract

Mice with inducible urothelial deletion of fibroblast growth factor receptor 2 (*ShhCreERT2;Fgfr2* ^*Fl/Fl*^) injured with cyclophosphamide had aberrant basal cell endoreplication and poor regeneration. The endoreplication correlated with an absence of phosphorylated (activated) ERK expression in urothelium. We assessed whether inhibiting ERK activity phenocopied the urothelial defects in injured *Fgfr2* mutant mice. We co‐administered cyclophosphamide and an ERK inhibitor (ERKi) systemically in mice and assessed general histology and immunofluorescence for various markers post injury. Since AKT also signals downstream of FGFR2, we assessed effects of an AKT inhibitor (AKTi) on cyclophosphamide injury. ERK knockdown did not affect urothelial injury or proliferation 24 h after cyclophosphamide. Conversely, ERK inhibition led to larger basal cell nuclei, more submucosal hemorrhage and attenuated uroplakin staining 3 days after injury versus vehicle‐treated mice. Compared to vehicle‐treated mice, ERKi‐treated mice had a trend for more Ki67^+^ urothelial cells and had statistically fewer phospho‐Histone H3^+^ cells normalized to Ki67 and higher basal cell DNA content, consistent with endoreplication 3 days after injury. Ten days after injury, ERKi‐treated mice still had signs of poor urothelial regeneration with absent or aberrant expression of differentiation markers and ectopic lumenal expression of keratin 14 (basal progenitor marker). Co‐administration of the AKTi led to no apparent urothelial defects 3 days after cyclophosphamide. Thus, ERK knockdown (but not AKT knockdown) leads to urothelial regenerative responses after cyclophosphamide reminiscent of *Fgfr2* mutant mice. Together, it appears that FGFR2 acts through ERK to prevent aberrant urothelial basal cell endoreplication and ensure normal regeneration after cyclophosphamide.

## Graphical abstract

Deletion of fibroblast growth factor receptor 2 in mouse urothelium leads to pathological endoreplication and poor repair after cyclophosphamide. Mice with knockdown of ERK have a similar pathological response to cyclophosphamide while knockdown of AKT causes no defects. Thus FGFR2 likely signals via ERK to suppress urothelial maladaptive responses to cyclophosphamide.

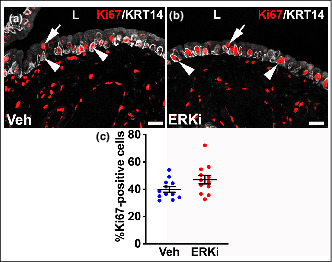

## 1 INTRODUCTION

Cyclophosphamide is an oxazophosphorine agent commonly used against solid tumors and lymphomas, as well as part of post‐transplantation immunosuppression after hematopoietic stem cell transplantation (Kaldor et al., 1995). Once administered, cyclophosphamide is metabolized by the liver to its metabolically active form, 4‐hydroxycyclophosphamide and further degrades to phosphoramide mustard and acrolein (Vlaovic & Jewett, 1999), the latter of which is highly toxic to bladder urothelial cells (Narla et al., 2020). Acute acrolein‐induced bladder injury can result in mild lower urinary tract symptoms and/or microscopic hematuria or lead to potentially life‐threatening hemorrhagic cystitis. Long‐term bladder complications from systemic cyclophosphamide/acrolein exposure include fibrosis and a dose‐dependent increased risk of cancer (Baker et al., 1987; Kaldor et al., 1995; Shirai, 1993; Vlaovic & Jewett, 1999).

Acute urothelial injury following cyclophosphamide administration is characterized by both urothelial cell necrosis and apoptosis followed by regeneration. Our laboratory has previously found that intraperitoneal (IP) injection of cyclophosphamide in mice leads to early nonapoptotic death of superficial cells followed by apoptosis in the intermediate and basal cell layers lasting up to 24 h (Narla et al., 2020). The urothelial regenerative response begins at 24 h after injury, characterized by proliferation of mainly keratin 14‐positive (KRT14^+^) basal cell progenitors. The regenerative response peaks at 3 days and diminishes thereafter. By 10 days post‐cyclophosphamide, we observed the return of most mature superficial cells in the bladder.

Our group and others have interrogated the roles of fibroblast growth factor (FGF) and FGF receptor (FGFR) signaling in blabber urothelial injury. To date, 19 signaling FGF ligands have been identified, which act via four signaling receptor tyrosine‐kinases FGFRs 1–4 that also consist of different isoforms (Ornitz & Itoh, 2015). Once activated, FGFRs lead to a cascade of phosphorylation events, including phosphorylation of the constitutively bound docking protein, fibroblast growth factor receptor substrate 2 alpha (FRS2α) that then triggers phosphorylation (activation) of ERK and AKT. Several years ago, a group demonstrated that systemic infusion of FGF7 in rats prior to cyclophosphamide led to more healthy appearing urothelium than vehicle‐treated rats, although the reasons for the better outcomes were unclear (Ulich et al., 1997). Our group did a follow‐up study showing that pretreatment with FGF7 largely blocked cyclophosphamide‐induced urothelial apoptosis and enhanced repair in mice (Narla et al., 2020). Furthermore, FGF7 appeared to activate its known receptor, FGFR2 (IIIb isoform) and FRS2α in urothelium, which correlated with the cytoprotection. Recently our group showed that FGF7‐induced cytoprotection appears to be via activation of AKT signaling, in that an AKT inhibitor blocked the cytoprotective effects of FGF7 and a pretreatment with a direct AKT agonist was sufficient to block apoptosis (Narla et al., 2022).

To assess the role of endogenous FGF7‐FGFR2 signaling in response to cyclophosphamide, we generated urothelial cell‐specific and inducible *Fgfr2* conditional mouse knockouts (*Fgfr2KO*) (Narla et al., 2021). After cyclophosphamide infusion, *Fgfr2KO* mice had urothelial regeneration defects starting at three post injury, leading to more hemorrhage in the stroma than controls. Moreover, the regenerative defects were characterized by higher numbers of urothelial cells engaged in the cell cycle (by Ki67 staining) that reflected pathological endoreplication, primarily in the basal cells. By 10 days after injury, the urothelium of controls had robust return of intermediate and superficial cells, while the *Fgfr2KO* mice still had poor urothelial cell regeneration. The pathological endoreplication in *Fgfr2KO* urothelium correlated with an absence of phosphorylated ERK staining 3 days post injury (which was present in controls), suggesting that the loss of ERK signaling was responsible for the defects. To interrogate the role of ERK signaling in preventing aberrant endoreplication and regeneration defects after injury, we assessed how a systemic ERK inhibitor (vs. an AKT inhibitor) affected the bladders of mice given cyclophosphamide in this current study.

## 2 MATERIALS AND METHODS

### 2.1 Mice

We used 2‐ to 3‐month‐old Female FVB/NJ mice for all experiments. All of the proposed mouse experiments were approved by the University of Pittsburgh Institutional Animal Care and Use Committee in accordance with the guidelines of the Association for Assessment and Accreditation of Laboratory Animal Care.

### 2.2 Experimental protocols in mice

To assess whether systemic inhibition of ERK led to urothelial regeneration defects after cyclophosphamide similar to what we saw in *ShhCreERT2;Fgfr2* ^*Fl/Fl*^ (*Fgfr2KO*) mice we administered a specific inhibitor of ERK1/2 (ERKi), SCH772984 (APEXBio, Cat # B5866), at 25 mg/kg or vehicle 0.9% sodium chloride (Ricca Chemical Company, Cat# 7210–16) by IP route starting at the time of injury (or sham) and then every 12 h for up to 60 h thereafter followed by tissue harvest at 1, 3, or 10 days after injury. To inhibit AKT signaling, we administered 40 mg/kg IP injections of the AKT inhibitor (AKTi), LY294002 (Selleckchem, Cat# S1105), dissolved in 2% dimethyl sulfoxide (DMSO) (Sigma‐Aldrich, Cat# D2438) or 2% DMSO alone (vehicle) starting at the time of injury (or sham) and then every 12 h for 60 h thereafter followed by tissue harvest at 3 days after injury. To induce injury, we administered a single dose of intraperitoneal cyclophosphamide 150 mg/kg (Sigma‐Aldrich, Cat# C7397) dissolved in phosphate‐buffered saline (PBS) or IP PBS alone (uninjured) concurrently with the first dose of ERKi, AKTi, or vehicle.

### 2.3 Histology, immunofluorescence, and TUNEL assays

For our histological experiments, we fixed isolated bladders in 4% paraformaldehyde (PFA), processed and embedded tissues in paraffin, and serially sectioned bladders at 6 μm. For general histology, we stained with hematoxylin and eosin (H&E). For immunofluorescence (IF), we dewaxed the paraffin‐embedded sections and subjected them to antigen retrieval in a pressure cooker for 15 min in Tris‐EDTA pH 9.0 buffer (TBS). We then blocked with normal donkey serum for 1 h at room temperature (RT). We then incubated sections overnight at 4°C with the following primary antibodies: anti‐keratin 20 (KRT20, Superficial cell marker) at 1:200 (Agilent Technologies, Cat# M7019, RRID:AB_2133718), keratin 14 (KRT14, basal cell marker) at 1:200 (BioLegend, Cat# 905301, RRID:AB_2565048), uroplakin 3a (UPK3, superficial cell and intermediate cell subset marker) at 1:200 (Santa Cruz Biotechnology, Cat# sc‐33,570, RRID:AB_2213486), phosphorylated ERK (pERK) at 1:200 (Cell Signaling Technology, Cat# 9100, RRID:AB_330741), phosphorylated AKT (pAKT) at 1:100 (Cell Signaling Technology, Cat# 4060, RRID:AB_2315049), (Ki‐67 (Ki67)), marker of proliferation/cell cycle activity, at 1:200 (R&D Systems, Cat# AF7649, RRID:AB_2687500), phospho‐Histone H3 (pHH3), marker of mitosis, at 1:100 (Cell Signaling Technology, Cat# 9701, RRID:AB_331535) and/or E‐cadherin at 1:200 (BD Biosciences, Cat# 610181, RRID:AB_397580). After washing in TBS, we incubated slides with the following secondary antibodies, Alexa Fluor 594 (Thermo Fisher, Cat# A‐21207, RRID:AB_141637), Alexa Fluor 488 (Thermo Fisher, Cat# A‐21202, RRID:AB_141607), and/or Alexa Flour 647 (Jackson ImmunoResearch Labs, Cat# 703–606‐155, RRID:AB_2340380), all at 1:500 for 2 h at RT, followed by washes. We stained nuclei with 4′6′‐diamidino‐2‐phenylindole (DAPI) (Sigma Aldrich, Cat# D1306). To assay for apoptosis, we performed TUNEL assays with an ApopTag Plus in situ Apoptosis Fluorescein Detection kit (EMD Millipore, Cat# S7111) according to the manufacturer's protocol. We imaged slides with a LeicaDM2500 fluorescence microscope (Leica Microsystems), a Leica Stellaris 5 Confocal microscope (Leica Microsystems), or a Zeiss LSM 710 confocal microscope (Carl Zeiss). We used a minimum of three bladders with two planes per bladder for each group (*N* = 6) for the samples one and 10 days after cyclophosphamide and four bladders with two planes per bladder for each group (*N* = 8) for our samples 3 days after cyclophosphamide for qualitative and quantitative assessments of staining (see figure legends for the *N* for each quantitative assay).

For DAPI quantification we obtained stacks of images using confocal microscopy with 1 μm steps. We analyzed 3D reconstructions with the Fiji distribution of ImageJ (software version 2.10/1.53c, http://Imagej.nih.gov/ij, NIH, Bethesda, MD, last accessed February 1, 2022). We then calculated the integrated density and volume of each nucleus and determined the ploidy by quantifying the intensity of DAPI within the nucleus. We set the average DAPI intensity of uninjured basal cells at *2n*. We calculated ploidy in 100 basal cells (25 nuclei from four bladders per group) in uninjured, injured vehicle‐treated mice, and injured ERKi‐treated mice (3 days after injury). We assessed urothelial proliferation rates (in percentages) 3 days after injury by counting Ki67^+^/DAPI^+^ urothelial cells (*N* = 12, including four bladders per group with three planes per bladder). Finally, we assessed the percentage of the lumenal urothelial surface expressing ectopic KRT14 staining 10 days after injury (*N* = 6, including three bladders with two planes per bladder). In all cases, the person quantifying expression was blinded to the treatment conditions of each sample.

### 2.4 Statistical analysis

For DAPI content in uninjured, injured vehicle‐treated, and injured ERKi‐treated mice, we applied mixed effect models (with random intercept for each mouse and 50 random bootstrap repetitions). For DAPI DNA quantification, we also used mixed effect model (DNA content a continuous outcome) and Fisher tests when comparing DNA content <2.5n, 2.5–4.5n, and >4.5n. To compare percentages of Ki67^+^ urothelial cells (Ki67^+^/DAPI^+^), percentages of pHH3^+^/Ki67^+^ urothelial cells, percentages of TUNEL^+^ urothelial cells (TUNEL^+^/DAPI^+^), and percentages of urothelial surface area with ectopic KRT14 staining, we performed Students *t*‐tests. We used a two‐sided hypothesis test for all analyses.

## 3 RESULTS

### 3.1 ERK inhibitor (ERKi) suppresses cyclophosphamide‐induced ERK activation urothelium

**FIGURE 1:**
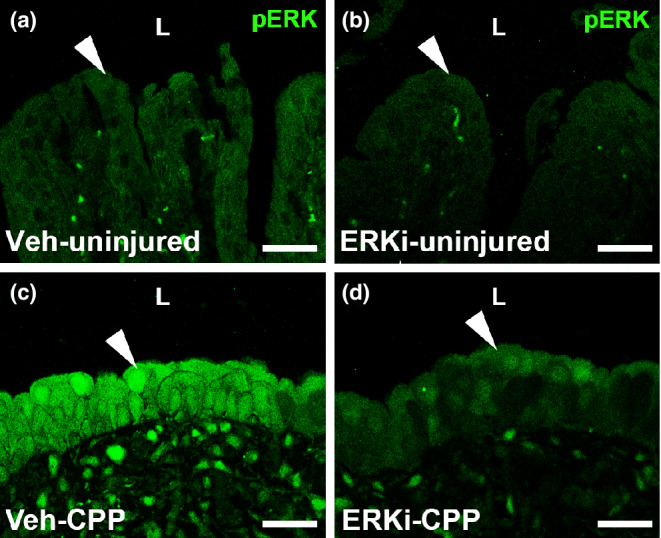
Representative image of phospho‐ERK (pERK) staining in vehicle (Veh) and ERK inhibitor (ERKi)‐treated mice 3 days after cyclophosphamide (CPP) or no injury. (a, b) IF for pERK (green) in uninjured mice shows no expression in the urothelium of those treated with vehicle (a, arrowhead) or ERK inhibitor (b, arrowhead). (c, d) Immunostaining for pERK in cyclophosphamide‐injured mice shows induction of urothelial expression in vehicle‐treated mice (c, arrowhead), which by comparison is largely attenuated in ERK inhibitor‐treated mice (d, arrowhead). “L” (a–d) = lumen. Scale bars (a–d) = 50 μm.

We sought to determine whether the apparent failure of *Fgfr2KO* mice to induce ERK signaling in urothelium after cyclophosphamide was responsible for the mutant urothelial regeneration defects after injury (Narla et al., 2021); thus, we treated wild‐type mice with a systemic ERKi. To ensure that the inhibitor prevented ERK activation in the urothelium after cyclophosphamide, we performed immunostaining against phosphorylated ERK (pERK) 3 days after injury. As expected uninjured mice treated with vehicle or ERKi had no obvious urothelial pERK expression (Figure 1). Immunostaining of injured mice showed robust pERK staining in the urothelium of the vehicle group but largely attenuated pERK staining in the injured ERKi group (Figure 1). Thus, the ERKi does appear to significantly inhibit the cyclophosphamide‐induced increase in urothelial pERK staining (ERK activation).

### 3.2 Mice given ERKi have similar degrees of urothelial injury and proliferation 1‐day post‐cyclophosphamide

**FIGURE 2:**
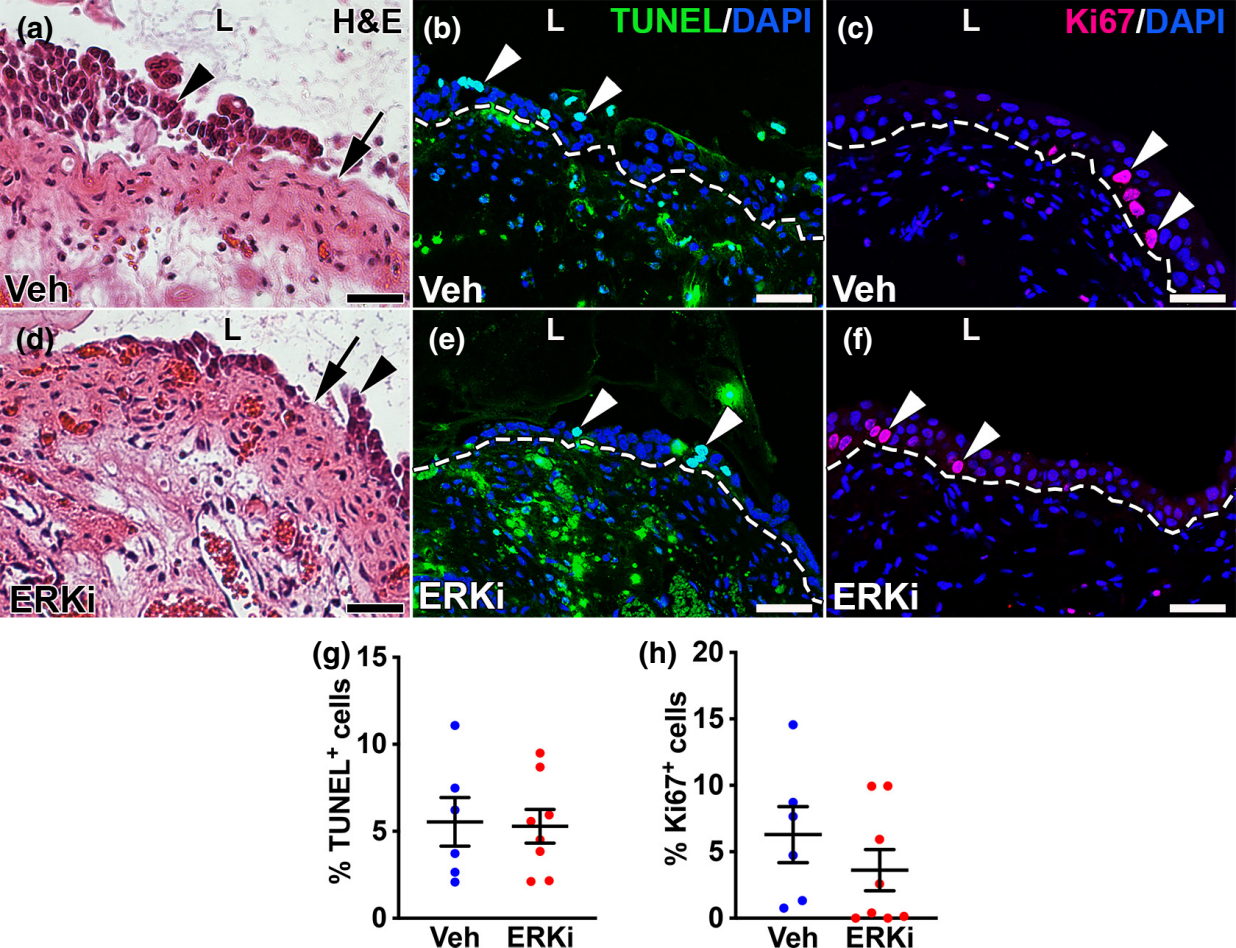
Representative images and graphs of urothelial injury and proliferation 1 day after cyclophosphamide injection in vehicle (Veh) and ERK inhibitor (ERKi)‐treated mice. (a, d) H&E stained images of vehicle‐treated (a) and ERKi‐treated bladders (d) show similar degrees of injury with sloughing urothelial cells (arrowheads) and denuded bladder lumenal surfaces (arrows). (b, e) TUNEL staining (green) in vehicle‐treated (b) and ERKi‐treated bladders (e) shows comparable numbers of apoptotic urothelial cells (arrowheads). (c, f) Ki67 staining (red) in vehicle‐treated (c) and ERKi‐treated bladders (f) shows similar numbers of proliferating urothelial cells (arrowheads). “L” (a–f) = lumen. Blue (b, c, e, f) = DAPI. Dashed line (b, c, e, f) = demarcation between urothelium and underlying lamina propria. Scale bars (a–f) = 50 μm. (g) Graph shows no statistical differences between mean percentages of TUNEL^+^/DAPI^+^ urothelial cells in the vehicle (*N* = 6) versus ERKi treated (*N* = 8) and injured cells (*p* = 0.88). Mean ± SEM. (h) Graph shows no statistical differences between mean percentages of Ki67^+^/DAPI^+^ urothelial cells in the vehicle (*N* = 6) versus ERKi treated (*N* = 8) and injured cells (*p* = 0.32). Mean ± SEM.

We next assessed whether systemic ERK inhibition affected urothelial injury or proliferation 24 h after cyclophosphamide. H&E stained images showed relatively equivalent severe urothelial damage 24 h after cyclophosphamide in the vehicle and ERKi‐treated samples, characterized by sloughing and denuding (Figure 2). TUNEL staining showed what appeared to be equivalent numbers of apoptotic urothelial cells in both the vehicle and ERKi‐groups (Figure 2); moreover, formal counts showed that mean percentages of TUNEL^+^ urothelial cells were similar between the two groups (vehicle group: 5.5% ± 1.4%, ERKi group: 5.3% 0.97%, *p* = 0.88) (Figure 2). Proliferation, assessed by Ki67 staining, also appeared comparable between vehicle and ERKi‐treated urothelium 24 h after cyclophosphamide (Figure 2); as with TUNEL assays, formal counts confirmed that mean percentages of KI67^+^ urothelial cells were similar between the two groups (vehicle: 6.3% ± 2.1%; ERKi: 3.6% 1.6%; *p* = 0.32) (Figure 2). These results parallel what we observed with *Fgfr2KO* mice in that control and mutant mice had similar numbers of apoptotic and proliferating cells 24 h after cyclophosphamide (Narla et al., 2021).

### 3.3 Mice given ERKi have evidence of urothelial regeneration defects 3 days post‐cyclophosphamide

**FIGURE 3:**
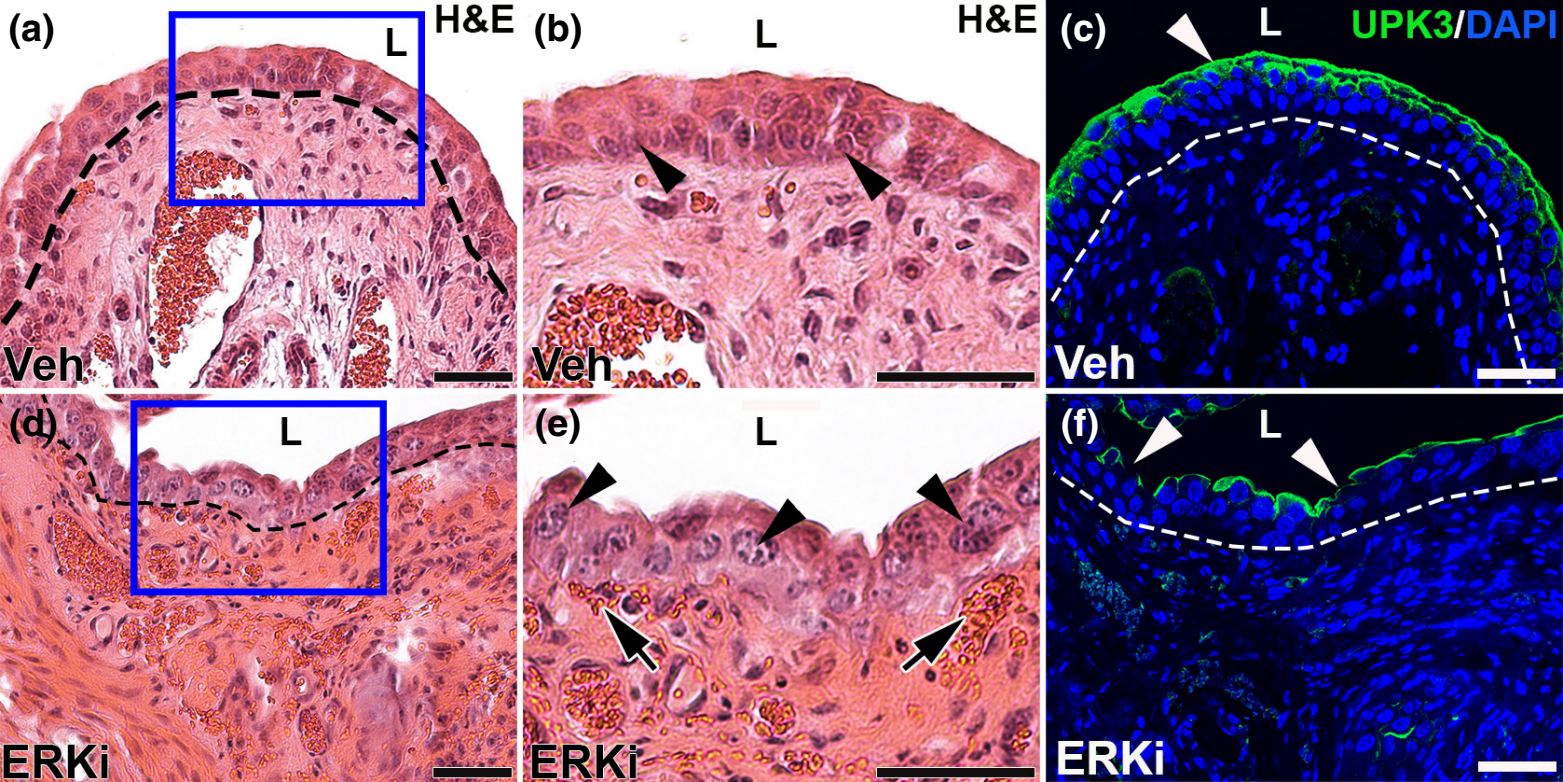
Representative images of urothelial injury 3 days after cyclophosphamide injection in vehicle (Veh) and ERK inhibitor (ERKi)‐treated mice. (a, d) H&E stained images of vehicle‐treated (a) and ERKi‐treated bladders (d) show similar relative thickness of urothelium 3 days after injury. (b, e) magnified regions (blue boxes) from a and d, respectively, show apparently more urothelial cell layers in the vehicle‐treated (b) than the ERKi‐treated bladders (e), many larger basal urothelial cell nuclei (arrowheads) in ERKi‐treated (e) than vehicle‐treated mice (e), and areas of submucosal hemorrhage in ERKi‐treated bladders (e, arrows) that are not present in vehicle‐treated mice (b). (c, f) IF for UPK3 (green) shows robust and continuous lumenal staining in vehicle‐treated mice (c) while ERKi‐treated bladders have attenuated or absent staining (f, arrowheads) 3 days after injury. “L” (a–f) = lumen. Blue (c, f) = DAPI. Dashed line (a, c, d, f) = demarcation between urothelium and underlying lamina propria. Scale bars (a–f) = 50 μm.

We next assessed how systemic suppression of ERK signaling affected urothelial regeneration 3 days post‐cyclophosphamide. H&E stained images revealed relatively similar urothelial thickness in injured mice‐treated vehicle or ERKi (Figure 3). Higher magnification of the H&E images, however, appeared to show fewer urothelial cell layers in the vehicle‐treated group versus the ERKi group and that the ERKi‐treated mice had larger basal cell nuclei than the former (Figure 3). In addition, the higher magnified H&E stained images showed regions of submucosal hemorrhage in the ERKi‐treated mice that were not present in the vehicle‐treated group (Figure 3). Immunostaining for KRT20 revealed the absence of mature KRT20^+^ superficial cells in both groups (not shown); however, staining for UPK3 in the same sections showed a strong UPK3 signal in those given vehicle versus attenuated or absent UPK3 staining in the ERKi group (Figure 3), consistent with the poor return of regenerating superficial cells in the latter. Thus, similar to *Fgfr2KO* mice (Narla et al., 2021), ERKi‐treated mice had urothelial regeneration defects 3 days' post‐cyclophosphamide, characterized by more hemorrhage, impaired superficial cell regeneration, larger basal cell nuclei, and fewer regenerating urothelial cells compared to vehicle‐treated mice.

**FIGURE 4:**
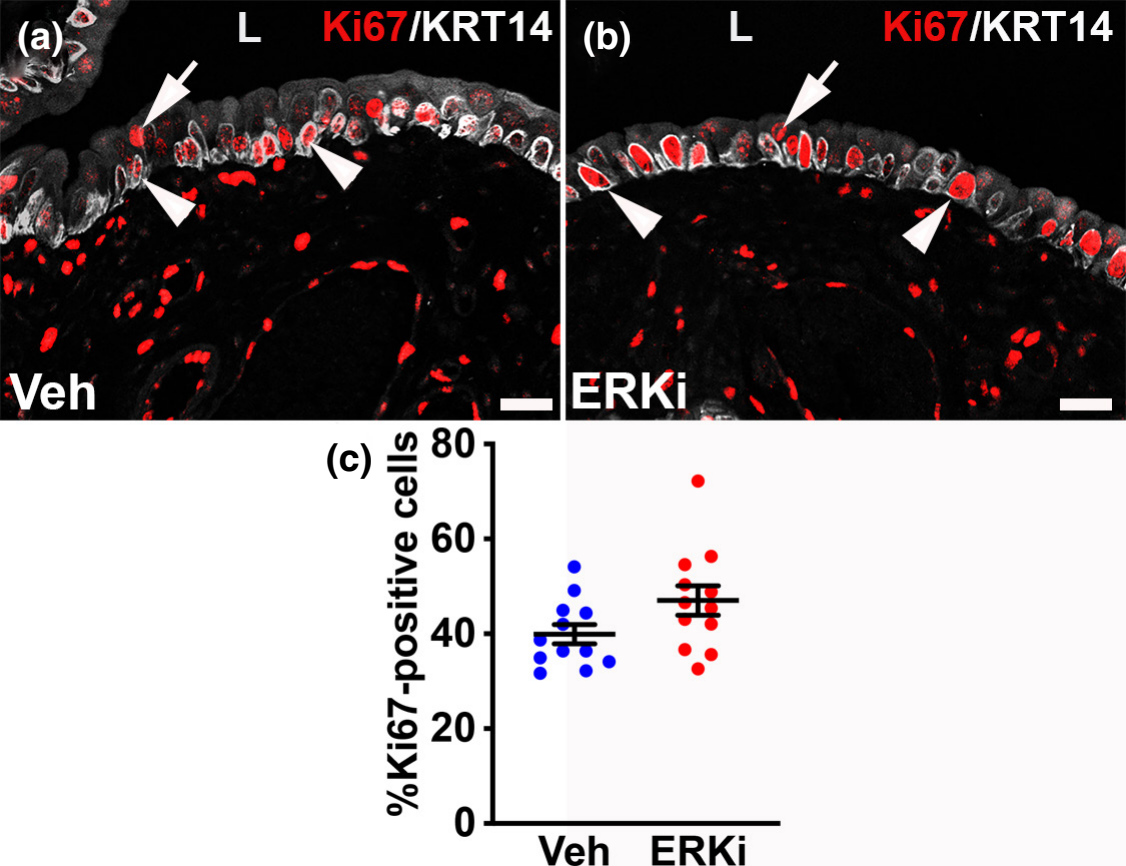
Representative images and graph of urothelial regeneration in vehicle (Veh) and ERK inhibitor (ERKi)‐treated mice 3 days after cyclophosphamide injury. (a) Double label IF for Ki67 (red) and KRT14 (white) shows that most of the Ki67^+^ urothelial cells (active in the cells cycle) in the vehicle‐treated and injured urothelial cells are KRT14^+^ (arrowheads) with few Ki67^+^ cells that are KRT14^−^ (arrow). (b) co‐IF for Ki67 and KRT14 in the ERKi‐treated and injured mice shows a similar pattern with more Ki67^+^/KRT14^+^ urothelial cells (arrowheads) than Ki67^+^/KRt14^−^ cells (arrow), although there appear to be more double‐positive cells, many with larger basal cell nuclei in the ERKi than the vehicle‐treated mice. “L” (a, b) = lumen. Scale bars (a, b) = 50 μm. (c) Graph shows a trend for higher mean percentages of Ki67^+^/DAPI^+^ urothelial cells in the ERKi versus vehicle‐treated and injured cells (*p* = 0.07). *N* = 12 in all groups. Mean ± SEM.

To further characterize the urothelial regenerative defects in the ERKi‐treated mice, we assessed immunostaining for Ki67 (proliferation/cell cycle activity marker) in combination with KRT14 (basal progenitor cell marker) 3 days after cyclophosphamide. As expected, most of the Ki67^+^ urothelial cells were also KRT14^+^ in both vehicle and ERKi‐treated mice (Figure 4). Similar to *Fgfr2KO* mice (Narla et al., 2021), however, ERKi‐treated and injured mice appeared to have larger Ki67^+^/KRT14^+^ cell nuclei than vehicle‐treated mice (Figure 4), correlating with the larger basal cell nuclei seen on the higher magnified H&E stained images (see Figure 3 above). In addition, ERKi‐treated mice appeared to have more Ki67^+^ urothelial cells than the vehicle‐treated (similar to *Fgfr2KO* vs. controls (Narla et al., 2021)). Direct counts of Ki67^+^ urothelial cells did reveal a trend for higher mean numbers of positive cells in the ERKi versus vehicle‐treated urothelium (Figure 4c, *p* = 0.07). Collectively, the findings in the ERKi group relative to the vehicle group are similar to what we observed in the *Fgfr2KO* mice versus control mice 3 days after cyclophosphamide (Narla et al., 2021).

### 3.4 Basal urothelial cells in mice treated with ERKi have apparent endoreplication 3 days after cyclophosphamide

**FIGURE 5:**
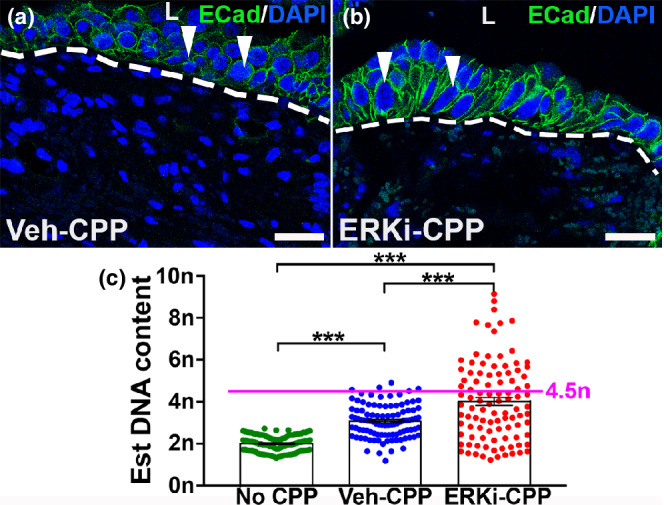
Representative images and graph of DAPI^+^ nuclei and DNA content 3 days after cyclophosphamide (CPP) injury in vehicle (Veh) and ERK inhibitor (ERKi)‐treated mice. (a, b) IF for E‐cadherin (green) with DAPI (blue) shows that compared to basal cell DAPI^+^ nuclei in vehicle‐treated mice (a, arrowheads), many basal cell DAPI^+^ nuclei in ERKi‐treated mice appear larger (b, arrowheads) 3 days post‐CPP. “L” (a, b) = lumen. Dashed line (a, b) = demarcation between urothelium and underlying lamina propria. Scale bars (a, b) = 25 μm. (c) Graph of DAPI^+^ basal cell DNA content showing a tight cluster around *2n* for uninjured urothelium, a range of *2*–*4.5n* for vehicle‐treated and injured urothelium and a range of *2–9n* for ERKI‐treated and injured urothelium. Injured vehicle‐treated urothelium have 5% of basal cell nuclei >*4.5n* while injured ERKi‐treated urothelium have 37% of basal cell nuclei >*4.5n* DNA content. Pink dashed line (c) = *4.5n* cutoff. *N* = 100 in all groups (four bladders with 25 cells per bladder). Mean ± SEM. ***p < 0.001 from mixed effect model or Fisher's exact test for cut off of *4.5n.*

The increased relative number of Ki67^+^ urothelial cells, larger sizes of KRT14^+^ basal cells, and fewer overall urothelial cell layers in ERKi‐treated and injured mice compared to vehicle‐treated mice, suggested that the former may have basal cell progenitors that were undergoing pathological endoreplication after injury, similar to *Fgfr2KO* mice (Narla et al., 2021). To test for this, we stained tissues for E‐cadherin to define cell borders and assessed DAPI intensity of individual basal cells by confocal microscopy to quantify DNA content. Using uninjured control basal cell mean DAPI intensity as a reference for cells that not proliferating (*2n*), we assessed injured mice given vehicle or given ERKi. We observed no binuclear basal cells in any groups (i.e., none of the groups had evidence of endomitosis in the basal cells). Compared to uninjured controls, which had very similarly sized basal nuclei (not shown), injured vehicle‐treated nuclei had a range of sizes including many similar to uninjured controls, but some that were larger (which is expected given that the injured‐vehicle group had cells undergoing proliferation, including DNA doubling at completion of S‐phase) (Figure 5). ERKi‐treated and injured mice had an even larger range of basal cell nuclei sizes, with many appearing much larger than injured vehicle‐treated mice (Figure 5). Formal quantification showed that uninjured control basal cell DAPI intensity clustered tightly around *2n*, whereas injured vehicle‐treated mice were mostly in the range of *2*–*4.5n* (Figure 5c). Moreover, injured ERKi‐treated animals had a wider distribution in basal nuclear content with many nuclei with >*4.5n* consistent with polyploidy (Figure 5c). Indeed, mean ploidy was also different among the three groups by mixed effect model (Figure 5). Fisher tests also revealed differences between all pairs of groups (*p* < 0.001); moreover, only 5% of injured controls were *>4.5n* versus 37% of the ERKi‐treated group. These data mimic we found when comparing DNA content in injured *Fgfr2KO* mice versus injured control mice and uninjured mice (Narla et al., 2021).

**FIGURE 6:**
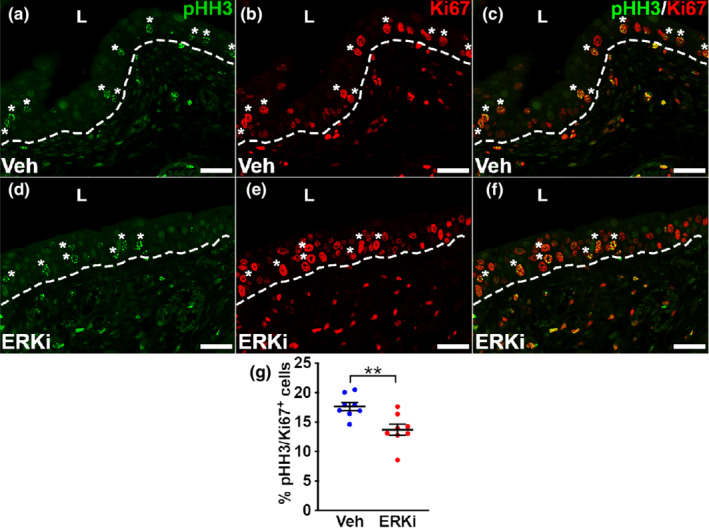
Images and graph of phospho‐histone H3 (pHH3) and Ki67 staining in urothelium 3 days after cyclophosphamide injury in vehicle (Veh) and ERK inhibitor (ERKi)‐treated mice. (a–f) co‐IF for pHH3 (green) and Ki67 (red) in vehicle (a–c) and ERKi‐treated (d–f) mice. (a–c) images showing vehicle‐treated mice with pHH3^+^ urothelial cells (a) that are all Ki67^+^ (b) as confirmed on merged staining (c). (d–f) images of ERKi‐treated mice show similar pattern, but relatively fewer pHH3^+^ cells (d) that are all K67^+^ (e) as confirmed on merged staining (f). Asterisks (a–f) = all pHH3^+^ cells. “L” (a–f) = lumen. Dashed line (a–f) = demarcation between urothelium and underlying lamina propria. Scale bars (a–f) = 50 μm. (g) Graph shows that the mean percentage of pHH3^+^ over total Ki67^+^ urothelial cells is higher in the vehicle‐treated group than that ERKi‐treated group. *N* = 8 in all groups. Mean ± SEM. **p < 0.01.

To further interrogate whether the ERKi‐treated urothelium was undergoing endoreplication (i.e. bypassing mitosis) relative to the vehicle‐treated mice 3 days after injury, we next performed co‐immunofluorescence for phospho‐Histone H3 and Ki67. Aurora kinases A and B phosphorylate serine 10 of Histone H3 at the start of mitosis, and as such pHH3 staining is specific for mitosis (Crosio et al., 2002), whereas Ki67 marks the entire cell cycle other than G0. Compared to vehicle‐treated mice, ERKi‐treated mice appeared to have fewer pHH3^+^ urothelial cells, which all co‐labeled with Ki67 in both groups as expected (Figure 6a–f). Formal quantification showed that the mean percentage of pHH3^+^/Ki67^+^ urothelial cells was approximately 30% higher in the vehicle‐treated mice versus the ERKi‐treated mice (17.7% ± 0.69% vs. 14.6% ± 0.95%, respectively, *p* < 0.01) (Figure 6g), consistent with the ERKi‐treated mice bypassing mitosis relative to the vehicle‐treated mice. Taken together with the DAPI quantification data, ERKi‐treated mice had evidence of pathological urothelial basal cell endoreplication 3 days after cyclophosphamide, similar to injured *Fgfr2KO* mice (Narla et al., 2021).

### 3.5 Mice given ERKi continue to have urothelial regeneration defects 10 days post‐cyclophosphamide compared to those given vehicle

**FIGURE 7:**
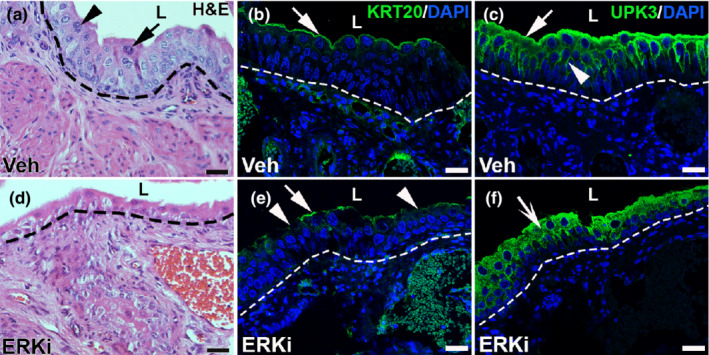
Representative images of urothelium in vehicle (Veh) and ERK inhibitor (ERKi)‐treated mice 10 days after cyclophosphamide. (a, d) H&E stained images of vehicle (a) and ERKi‐treated bladders (d) show more regenerating urothelial cell layers and more mature superficial cells with large nuclei (arrow) or two nuclear moieties (arrowhead) in vehicle‐treated mice (a) than ERKi‐treated mice (d) 10 days after injury. (b, e) IF for KRT20 (green) shows nearly complete return of continuous lumenal staining in vehicle‐treated mice (b, arrow), while the ERKi‐treated mice have some regions of staining (e, arrow) are interrupted by many areas of lost or attenuated staining (e, arrowheads). (c, f) IF for UPK3 (green) in vehicle‐treated mice (c) shows strong linear surface staining (arrow) and sharp basolateral staining (arrowhead), while in ERKi‐treated mice (f), the staining is more diffuse and appears to distributed largely in the cytoplasm (concave arrow). “L” (a–f) = lumen. Blue (b, c, e, f) = DAPI. Dashed line (a–f) = demarcation between urothelium and underlying lamina propria. Scale bars (a–f) = 50 μm.

**FIGURE 8:**
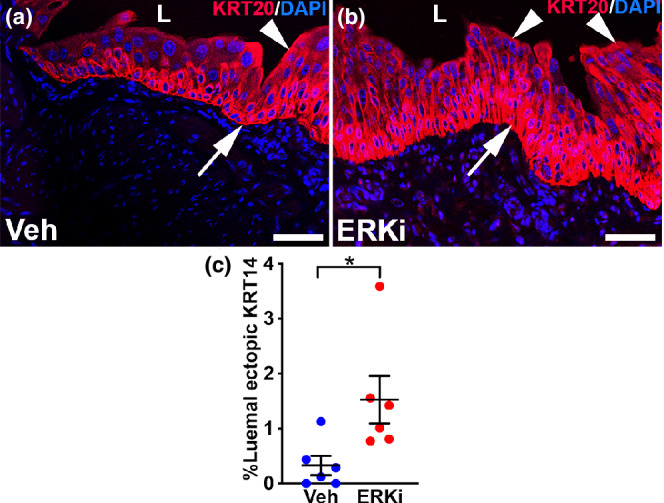
Representative images and graph of ectopic KRT14 expression in vehicle (Veh) and ERK inhibitor (ERKi)‐treated mice 10 days after cyclophosphamide. (a, b) IF for KRT14 (red) shows strong basal‐layer staining (arrows) in vehicle‐treated (a) and ERKi‐treated mice (b) and with some ectopic lumenal expression (arrowheads) that appears more widespread in vehicle‐treated (a) than ERKi‐treated mice (b). “L” (a, b) = lumen. Blue (a, b) = DAPI. Scale bars (a, b) = 50 μm. (c) Graph shows that the mean percentage of lumenal ectopic KRT14 expression is higher in the ERKi‐treated group than the vehicle‐treated group. *N* = 6 in all groups. Mean ± SEM. *p < 0.05.

**FIGURE 9:**
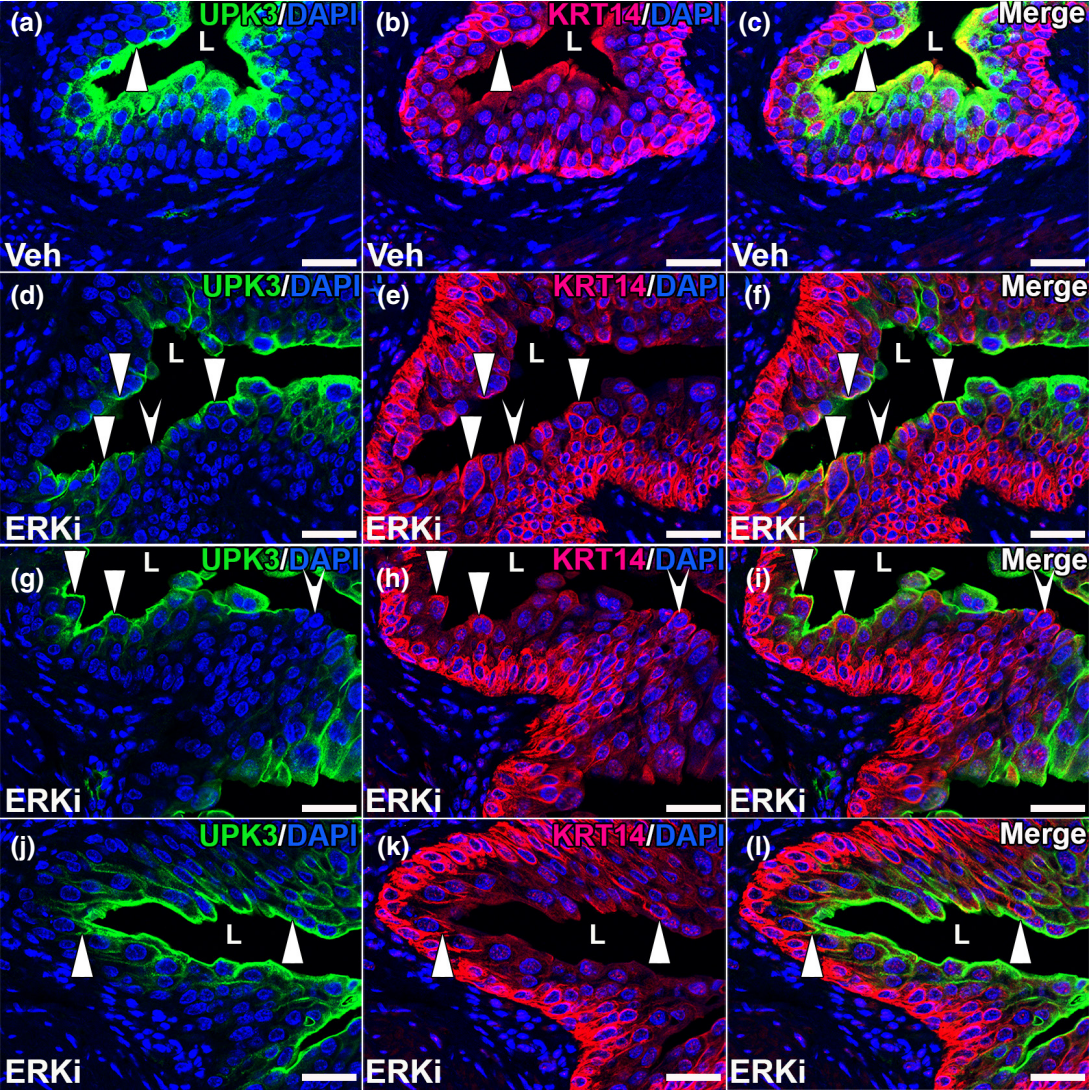
Representative images of UPK3 and KRT14 co‐expression in many lumenal urothelial cells in vehicle (Veh) and ERK inhibitor (ERKi)‐treated mice 10 days after cyclophosphamide. (a–l) co‐IF for UPK3 (green) and KRT14 (red). (a–c) a vehicle‐treated mouse has a UPK3^+^ lumenal cell (a, arrowhead) that is also KRT14^+^ (b, arrowhead) as confirmed on the merged image (c, arrowhead). (d–l) three separate ERKi‐treated mice (d–f, g–i, j–l, respectively), show many lumenal UPK3^+^ cells that are also KRT14^+^ (arrowheads) and a few lumenal UPK3^−^ cells that are KRT14^+^ (concave arrowheads). “L” (a–l) = lumen. Blue (a–l) = DAPI. Scale bars (a–l) = 50 μm.

We then assessed the effects of ERKi treatment on urothelial regeneration 10 days after cyclophosphamide infusion. H&E staining showed that while the vehicle‐treated mice had evidence of ongoing urothelial cell regeneration, they also demonstrated return of many polyploid mature superficial cells (Figure 7). In comparison, the ERKi‐treated mice had fewer urothelial cell layers (consistent with poor regeneration) and few polyploid superficial cells (Figure 7). Immunostaining for KRT20 (mature superficial cell marker) showed largely robust staining in vehicle‐treated mice, but attenuated or absent signal in ERKi‐treated mice 10 days post injury, consistent with poor return of superficial cells in the latter (Figure 7). Similarly, immunostaining for UPK3 (marker of intermediate cell subset and superficial cells) showed strong linear lumenal and basolateral staining in outer urothelial cells in the vehicle group, but more aberrant and diffuse, cytoplasmic staining in the ERKi‐treated group 10 days post injury, consistent with poor repair in the latter (Figure 7). We then performed immunofluorescence for KRT14 and noted many more KRT14^+^ cells overall in the ERKi group versus the vehicle group (Figure 8). Moreover, the ERKi‐treated group appeared to have larger numbers of ectopic lumenal KRt14^+^ cells than the vehicle‐treated group (Figure 8), which we confirmed by quantifying the percentage of the lumenal surface that was KRT14^+^ (vehicle group: 0.33% ± 0.17%, ERKi group: 1.53% ± 0.43%, *p* < 0.05). (Figure 8). We also found that most of the KRT14^+^ cells at the lumenal surface co‐expressed UPK3 in both the vehicle and ERK‐treated groups, which is another testament to aberrant urothelial repair that is exacerbated in the ERKi‐treated groups (Figure 9). Thus, similar to *Fgfr2KO* mice, ERKi‐treated mice had ongoing regeneration defects 10 days after cyclophosphamide compared to vehicle‐treated mice (Narla et al., 2021).

### 3.6 Inhibition of AKT does not result in aberrant regenerative defects in urothelium after cyclophosphamide injury

**FIGURE 10:**
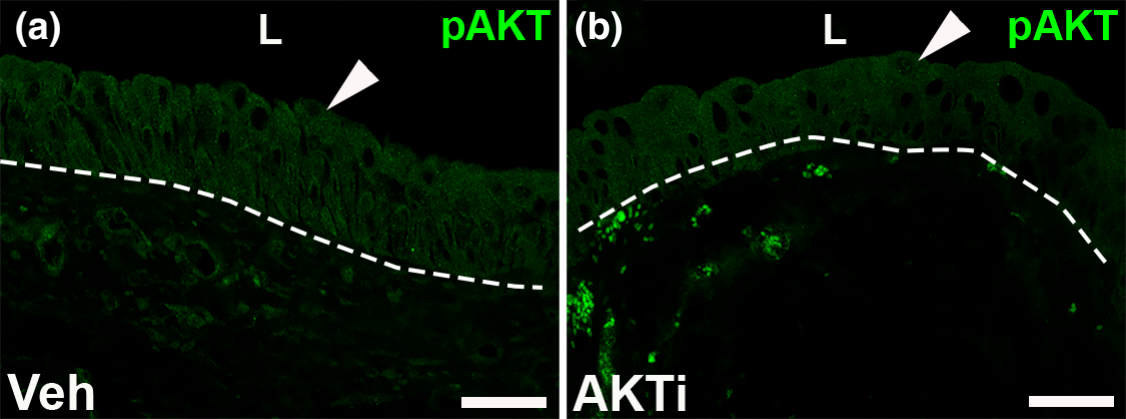
Representative image of phosphorylated‐AKT (pAKT) staining in vehicle (Veh) and AKT inhibitor (AKTi)‐treated mice 3 days after cyclophosphamide (CPP) or no injury. (a, b) immunostaining for pAKT (green) shows no obvious urothelial expression (arrowheads) in either the vehicle‐treated (a) or AKTi‐treated mice (b). “L” (a–f) = lumen. Dashed line (a, b) = demarcation between urothelium and underlying lamina propria. Scale bars (a, b) = 50 μm.

Since activation of AKT is another of the major signaling arms of FGF signaling, we sought to determine if systemic inhibition of AKT activity would lead to aberrant urothelial regeneration defects similar to *Fgfr2KO* mice. To that end, we administered a well‐known AKT inhibitor that we have shown previously can largely abrogate systemic FGF7‐induced increases in urothelial pAKT staining (Narla et al., 2020). Thus, similar to the ERKi experiments, we co‐administered the AKTi and cyclophosphamide and continued giving the inhibitor every 12 h up until harvest 3 days post injury. Immunostaining for pAKT in vehicle‐ and AKTi‐treated mice showed no obvious urothelial staining (Figure 10) (consistent with our previous observations that cyclophosphamide alone does not appear to upregulate urothelial pAKT expression 3 days after injury (Narla et al., 2020)).

**FIGURE 11:**
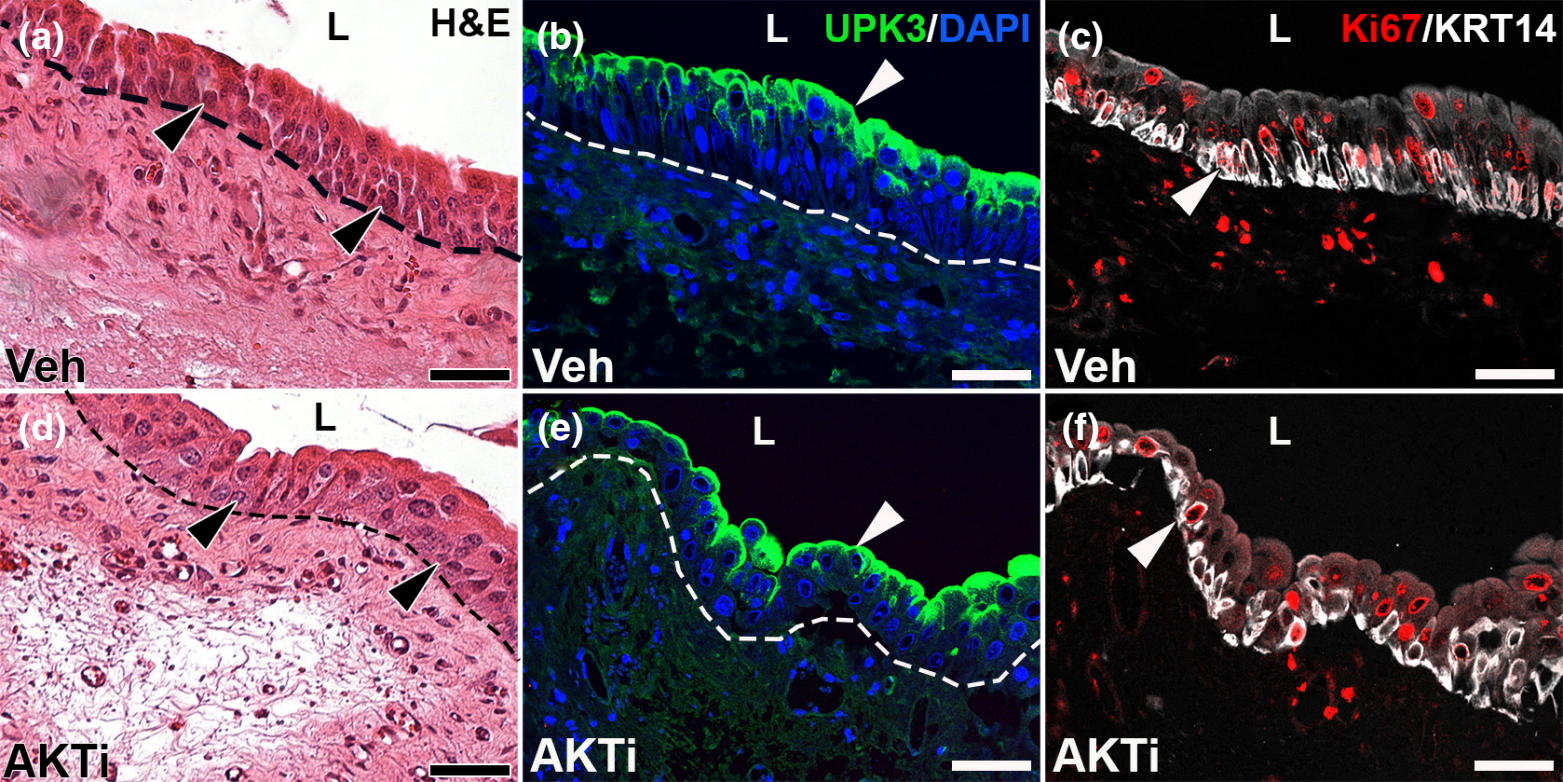
Representative images of urothelial injury 3 days after cyclophosphamide injection in vehicle (Veh) and AKT inhibitor (AKTi)‐treated mice. (a, d) H&E stained images of vehicle‐treated (a) and AKTi‐treated bladders (d) show similar relative thickness of the urothelium and small basal cell nuclei (arrowheads) 3 days after injury. (b, e) IF for UPK3 (green) shows robust and continuous lumenal staining (arrowheads) in both vehicle‐treated (b) and AKTi‐treated mice (e) 3 days after injury. (c, f) double label IF for Ki67 (red) and KRT14 (white) in vehicle‐treated (c) and AKTi‐treated mice (f) shows similar numbers of proliferating urothelial cells and KRT14^+^ basal cells, many of which co‐label with Ki67 and appear to be similar in size (arrowheads). “L” (a–f) = lumen. Blue (b, e) = DAPI. Dashed line (a, b, d, e) = demarcation between urothelium and underlying lamina propria. Scale bars (a–f) = 50 μm.

We next assessed the effects of the AKTi on urothelial regeneration 3 days after injury. H&E stained images revealed relatively similar thickness and small basal cell nuclei in both groups (Figure 11). In addition, immunostaining for UPK3 showed similar robust staining at the lumenal urothelial surface in vehicle‐treated and AKTi‐treated mice (Figure 11). Finally, double‐label immunofluorescence for Ki67 and KRT14 showed similar numbers and sizes of Ki67^+^ and KRT14^+^ urothelial cells (including double‐positive cells) in both groups. Thus, knockdown of AKT does not appear to cause obvious urothelial regeneration defects after cyclophosphamide, making it likely that the loss of ERK signaling in *Fgfr2KO* mice is indeed responsible for the defective urothelial regeneration in the mutants.

## 4 DISCUSSION

In the current study, we observe that systemic administration of SCH772984, an ERK inhibitor, is able to largely suppress cyclophosphamide‐induced increases in urothelial pERK expression 3 days after injury. We observed no differences in urothelial injury or proliferation 24 h after cyclophosphamide in the vehicle and ERK inhibitor‐group (just as we noted comparable urothelial injury and proliferation in *Fgfr2* conditional mutants and controls 24 h after cyclophosphamide (Narla et al., 2021)). Furthermore, we observed defects in urothelial regeneration in the ERKi‐treated mice at 3 days post injury, characterized by reduced uroplakin staining, submucosal hemorrhage, and larger basal cell nuclei. We noted a trend for increased numbers of Ki67^+^ cells, particularly in KRT14^+^ cells in the ERKI‐group, consistent with increased cell cycle activity compared to vehicle‐treated mice. That increased cell cycle activity correlated with higher DNA content in the basal cells consistent with aberrant endoreplication, which is likely the reason for the poor regeneration after injury. The ratio of pHH3^+^/Ki67^+^ urothelial cells was approximately 30% higher in the vehicle‐treated mice versus the ERKi‐treated mice, consistent with the latter bypassing mitosis relative to the former. The regeneration defects in the ERKi‐treated mice persisted by 10 days after injury, with reduced urothelial cell layers, evidence of fewer mature superficial cells, and more ectopic lumenal KRT14^+^ cells. Most of the ectopic KRT14^+^ cells were also UPK3^+^, which is not seen in uninjured urothelium. All of these defects in the ERKi‐treated and injured mice are also seen in injured mice with conditional deletion of *Fgfr2* in the urothelium, with ectopic lumenal KRT14^+^ cells noted even 6 months after injury in both mutant and control mice (Narla et al., 2021). The similarities in phenotypes strongly suggest that intact FGFR2‐ERK signaling is required to suppress pathological endoreplication and promote normal urothelial regeneration after cyclophosphamide.

Other observations support that the loss of ERK activity downstream of FGFR2 leads to the aberrant basal cell endoreplication in *Fgfr2KO* mice (Narla et al., 2021). The timing of ERK activation in urothelium after cyclophosphamide injury correlates with the onset of endoreplication in *Fgfr2KO* mice 3 days after injury. In prior work, we observed no obvious increase in pERK signal in urothelium of wild‐type mice 1 day after cyclophosphamide (Narla et al., 2020); however, in both the current study and another, we noted strong sustained pERK staining in urothelium 3 days after injury (Narla et al., 2021). A different group also observed no increases in pERK by bladder western blot or immunofluorescence in urothelium of rats until at least 48 h after injury, which persisted for 10 days (Corrow & Vizzard, 2007). Finally, a large body of work has shown that sustained ERK signaling can suppress cell cycle progression and inhibit cyclin‐cyclin‐dependent kinase complexes that promote endoreplication (Chambard et al., 2007; Gandarillas et al., 2018; LaBaer et al., 1997; Park et al., 2000). Thus, it appears that endogenous FGFR2 signaling is required to drive ERK activity in urothelium to suppress basal cell endoreplication after cyclophosphamide.

While AKT is another major signaling arm downstream of FGFR2, it appears to have no obvious role in suppressing urothelial endoreplication to allow for a normal regenerative response. The timing of pAKT activation in urothelium after cyclophosphamide appears to peak prior to the onset of basal cell endoreplication in basal cells. In prior work, we determined that cyclophosphamide leads to an increase in pAKT urothelial expression by 24 h after injury (Narla et al., 2020). However, in this study and another, we did not note any obvious pAKT staining in urothelium by 3 days after injury (Narla et al., 2021). Similarly, another group noted that cyclophosphamide induced increased pAKT levels in whole bladder western blots and by immunofluorescence in urothelium of rats by 4 h after injury, which vastly diminished by 48 h (Arms & Vizzard, 2011). Moreover, co‐activation of AKT and ERK had been shown to suppress the inhibitory effects of ERK on the cell cycle (Chambard et al., 2007), making it unlikely that AKT signaling has any role in suppressing endoreplication. Finally, we found that direct suppression of AKT with LY294002 (using a dosing strategy that we know blocks induction of pAKT in urothelium in other conditions (Narla et al., 2022)) had no obvious effects on urothelial regeneration after cyclophosphamide. Together, these data support that it is indeed ERK and not AKT signaling downstream of FGFR2 that is required to suppress basal cell endoreplication to ensure a normal urothelial regenerative response after cyclophosphamide.

Our findings from these and prior studies may have clinical implications. In our prior study, we noted that deletion of even just one *Fgfr2* allele in the urothelium led to aberrant basal cell endoreplication and poor urothelial cell regeneration after cyclophosphamide (albeit less than in the complete knockouts) (Narla et al., 2021). Moreover, mice that are globally heterozygous for *Fgfr2* are also phenotypically normal (Arman et al., 1998; Xu et al., 1998); thus, single‐gene deletions or polymorphisms in FGF7/FGF10/FGFR2IIIb family signaling in humans may not lead to any outward disease, but could predispose them to significant urothelial regeneration defects if exposed to cyclophosphamide. In addition, several disease‐causing mutations/variants in the FGF7/10/FGFR2IIIb family have been described (Belov & Mohammadi, 2013; Itoh & Ornitz, 2011; Zinkle & Mohammadi, 2019) and as such, these individuals could be at heightened risk for life‐threatening hemorrhagic cystitis or urothelial cancer risk later in life if exposed to cyclophosphamide. Finally, mice with complete deletion of *Erk1* are viable without outward signs of disease (Pages et al., 1999), and as such humans with loss of ERK1 or that are hypomorphic for ERK1 or ERK2 could be at high risk of these same urothelial complications after cyclophosphamide. Thus, as the use of exome sequencing in patients becomes more highly utilized, identification of mutations or variants in these aforementioned genes may guide whether clinicians would treat patients with cyclophosphamide (or reduce the dose) and/or would screen more carefully for long‐term urothelial cancer risks. Finally, as KRT14^+^ cell cycle activation occurs after many other types of urothelial injury, such as spinal cord injury (Kullmann et al., 2017), radiation cystitis (Jaal & Dorr, 2010), and urinary tract infections (Colopy et al., 2014; Mysorekar et al., 2009), people with mutations or variants in FGF7/FGF10/FGFR2IIIb or ERK1/2 families could have increased risks for abnormal urothelial regeneration after these insults.

Limitations of the study include that the mouse model of injury does not exactly mimic what happens in humans. Most notably, we are using approximately 10‐times the dose of cyclophosphamide (based on body weight) than is typically used for a single dose in people (Cunningham et al., 2013). That said, hemorrhagic cystitis typically occurs within 24–48 h after cyclophosphamide in people (George, 1963). In addition, a study using cystoscopy and bladder biopsies in patients 24 h after receiving ifosfamide (also metabolized to acrolein) noted urothelial injury in the majority of patients (Lima et al., 2007). Thus, the onset of urothelial injury in humans appears similar to what we see in our mouse model. Other potential limitations include that the systemic ERK and AKT inhibitors likely have off target effects. While systemic AKT and ERK inhibitors can be given transurethrally (Arms & Vizzard, 2011; Corrow & Vizzard, 2007), we did not use this mode of delivery as we were concerned that we could inflict additional injury to the bladders by filling them and clamping them for the 30 minutes that is required to allow for the inhibitors to traverse the urothelium. Finally, we noted no obvious side effects of systemic delivery of either inhibitor.

## AUTHORS' CONTRIBUTIONS

Sridhar Narla and Joanne Duara were involved in study design, conducting experiments, analysis, and writing and editing of the manuscript. Daniel Bushnell, Jacqueline Holden, and Katherine Pfister were involved in conducting experiments. Mehdi Nouraie carried out statistical analyses. Peter Lucas carried out analysis and validation. Sunder Sims‐Lucas carried out supervision and analysis. Carlton Bates carried out conceptualization, project administration, supervision, analysis, writing, reviewing, and editing of the manuscript.

## CONFLICT OF INTEREST

The authors have no conflicts to declare.
